# Cognitive cartography of mammalian brains using meta-analysis of AI experts

**DOI:** 10.64898/2025.12.01.691701

**Published:** 2026-10-19

**Authors:** Andrea I Luppi, Hana Ali, Zhen-Qi I Liu, Filip Milisav, Arianna Sala, Jitka Annen, Alessandro Gozzi, Danilo Bzdok, Bratislav Misic

## Abstract

No individual mind can fully grasp the complexity of modern neuroscientific literature. Where human experts flag, the latest AI models excel. Here we show how frontier large language models can systematically and quantitatively summarise literature-wide neuroscientific knowledge about the cognitive functions and dysfunctions associated with each brain region. Meta-analysis of these AI experts reveals structure-function mappings to which existing meta-analytic frameworks are blind, demonstrated by causal manipulations including lesions and intracranial stimulation. It also unlocks the possibility of extending quantitative literature meta-analysis and decoding of brain maps to other model organisms beyond human. As proof-of-concept, we integrate LLM meta-analysis with species-specific transcriptomics and connectomics in human, macaque, and mouse, discovering that the human molecular circuit for cognition is evolutionarily conserved. Altogether, literature synthesis with AI experts can fundamentally catalyze neuroscientific discovery by overcoming the barrier of data aggregation from heterogeneous studies, finally bringing together a scattered literature to identify latent insights across disparate subfields, modalities, and species.

